# APE1 inhibition-promoted pyroptosis triggers T-cell infiltration and enhances anti-tumor immunity in NSCLC

**DOI:** 10.1016/j.gendis.2025.101813

**Published:** 2025-08-14

**Authors:** Fan Yang, Kaili Long, Yannan Qi, Yuwei Dai, Feiyan Pan, Lingfeng He, Lindong Yang, Zhigang Guo, Zhigang Hu

**Affiliations:** a Jiangsu Key Laboratory for Molecular and Medical Biotechnology, College of Life Sciences, Nanjing Normal University, Nanjing, Jiangsu 210023, China; b Department of Obstetrics and Gynecology, Jinling Hospital, School of Medicine, Nanjing University, Nanjing, Jiangsu 210018, China

Pyroptosis, a form of pro-inflammatory programmed cell death mediated by gasdermin (GSDM) proteins, has been shown to synergize with immune checkpoint inhibitors to improve tumor eradication.^1^ The key regulators of pyroptosis are inflammasomes, particularly NOD-, LRR- and pyrin domain-containing protein 3 (NLRP3), which can be activated by cytoplasmic mitochondrial DNA.^2^ Our previous studies identified apurinic/apyrimidinic endonuclease 1 (APE1), a DNA repair protein, as a key factor in inducing pyroptosis in non-small cell lung cancer (NSCLC) upon its deficiency.^3^ However, the molecular mechanism by which APE1 inhibition leads to pyroptosis in NSCLC remains to be elucidated. To investigate the mechanism of APE1-mediated cell pyroptosis, we used specific shRNA to construct APE1 knockdown cells (hereafter referred to as APE1-KD) based on two human NSCLC cell lines (A549 and NCI-H460) and a murine LLC cell line (Fig. S1A and S1B). The indicative pyroptotic morphology was detected in these APE1-KD cells (Fig. 1A; Fig. S1C). It was found that the release of pro-inflammatory cytokines, including interleukin 18 (IL-18), interferon-gamma (IFN-γ), and IL-2, and chemokines, including C-C motif chemokine ligand 5 (CCL5) and C-X-C motif chemokine ligand 10 (CXCL10), was increased in the APE1-KD cells (Fig. 1B; Fig. S1D–S1H). Additionally, the lactate dehydrogenase (LDH) level was higher in the APE1-KD groups than in the control group (Fig. S1I). The APE1-KD tumor cells also showed an incomplete cell membrane as indicated by Hoechst/propidium iodide staining (Fig. 1C; Fig. S1J). Mechanistically, APE1 depletion specifically activated the caspase-3–GSDME pyroptosis pathway, without affecting GSDMA, GSDMB, GSDMC, GSDMD, or caspase-1 (Fig. 1D; Fig. S1K and S1L). To further explore the mechanism by which APE1 depletion leads to pyroptosis in NSCLC cells, RNA sequencing (GEO: GSE220807) was performed to investigate the potentially regulated signaling pathway. Gene Set Enrichment Analysis (GSEA) revealed that the pathways associated with DNA binding were enriched in the APE1-KD cells (Fig. 1E). Consistently, the APE1-KD tumor cells showed higher levels of cytoplasmic dsDNA compared with the wild-type (WT) cells (Fig. S2A). Previous studies have identified that APE1 inhibition leads to an increase in the amount of mtDNA copy number in tumor cells.^4^ Using quantitative PCR to detect mtDNA levels by targeting the D-loop region (the origin of mtDNA replication), we found that the expression of cytoplasmic mtDNA in the APE1 depletion cells was higher than that in the control cells (Fig. S2B). To determine whether APE1 inhibition promotes pyroptosis through cytoplasmic mtDNA accumulation, we depleted mtDNA, which resulted in reduced caspase-3 activation and GSDME cleavage (Fig. 1F–H; Fig. S2C and S2D).

**Figure 1:**
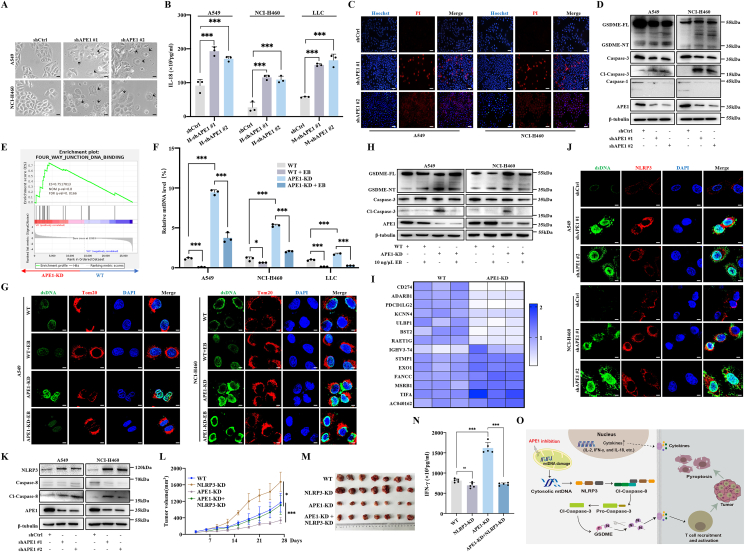
Targeting APE1 promotes cell pyroptosis by activating the NLRP3–caspase-3–GSDME pathway. **(A)** Tumor cell morphology was observed by transplantation with short hairpin RNA-mediated APE1. The black arrows indicate cell pyroptosis. Scale bar: 50 μm. **(B)** ELISA for IL8 release in the supernatants of A549, NCI-H460, and LLC cells. **(C)** Hoechst33342/propidium iodide (PI) fluorescent staining assay for detecting A549 and NCI-H460 cells' membrane integrity. Scale bar: 50 μm. **(D)** Western blots for cell pyroptosis protein levels in the A549 and NCI-H460 cells, including GSDME, caspase-3, and caspase-1. **(E)** Gene Set Enrichment Analysis (GSEA) showed the gene enrichment of APE1 knockdown in the NCI-H460 cells. **(F)** Detection of mtDNA expression in the A549, NCI-H460, and LLC cells treated with 10 ng/μL EtBr (EB) after 4 days. **(G)** Immunofluorescence detection of intracellular dsDNA co-localization with Tom20 in the A549 and NCI-H460 cells treated with 10 ng/μL EB after 48 h. Scale bar: 50 μm. **(H)** Western blots for cell pyroptosis protein levels in the A549 and NCI-H460 cells treated with 10 ng/μL EB after 4 days, including caspase-3 and GSDME. **(I)** Heat map of the gene enrichment of APE1 knockdown in the NCI-H460 cells. **(J)** Immunofluorescence detection of intracellular dsDNA co-localization with NLRP3 in the A549 and NCI-H460 cells. Scale bar: 10 μm. **(K)** Western blots for cell pyroptosis protein levels in the A549 and NCI-H460 cells, including NLRP3 and caspase-8. **(L)** The tumor volume (mm^3^) change trend of mice in the two different treatment groups. **(M)** Photographs of mouse tumors. **(N)** ELISA results of IFN-γ secretion levels in mouse blood. **(O)** The diagram illustrates that the inhibition of APE1 promotes tumor cell pyroptosis. In the proposed model, inhibition of APE1 in tumor cells activated the NLRP3–caspase-8 pathway by releasing mtDNA into the cytoplasm. Furthermore, activation of the casapse-3–GSDME pathway promotes tumor cell pyroptosis. The cell membrane is destroyed when tumor cells undergo pyroptosis. Tumor cells release inflammatory factors and chemokines, which further recruit and activate immune cells, and improve the tumor microenvironment to enhance the killing effect of immune cells on tumor cells. Data were presented as mean ± standard deviation of at least three independent experiments. ns, no significance; ∗*P* < 0.05; ∗∗∗*P* < 0.001.

RNA sequencing analysis revealed an up-regulation of genes associated with immune activation and a down-regulation of genes related to immune suppression in the APE1-KD NCI-H460 cells (Fig. 1I). Among these genes, several genes involved in activation of NLRP3 pathways were identified, including short transmembrane mitochondrial protein 1 (STMP1). These results implicated NLRP3 in the APE1 depletion-induced pyroptosis-mediated immune response in tumor cells. Consistently, confocal laser assay showed that NLRP3 co-localized with dsDNA in all three kinds of the APE1-KD NSCLC cells (Fig. 1J; Fig. S3A). By contrast, there were no significant changes in the absent in melanoma 2 (AIM2) and cyclic GMP-AMP synthase (cGAS)–stimulator of interferon gene (STING) pathways in the APE1-KD tumor cells compared with the control cells (Fig. S3B). The sensors and key markers in the NLRP3 pathway, including NLRP3, caspase-8, and IL-1α, were all up-regulated in the APE1-KD cells (Fig. 1K; Fig. S3C and S3D). These results demonstrate that APE1 depletion induces the accumulation of cytosolic mtDNA, which may activate the NLRP3–caspase-8 pathway. Previous studies have shown that NLRP3 preferentially senses oxidized mtDNA, rather than intact mtDNA, in the cytoplasm.^5^ APE1, a key enzyme in mitochondrial base excision repair, is required to resolve oxidative DNA lesions such as 8-oxoG (Fig. S3E). Similar to flap endonuclease-1 (FEN1), APE1 deficiency may impair mtDNA repair, leading to the cytosolic release of oxidized mtDNA and subsequent immune activation.^5^ We also found that depleting mtDNA in the APE1-KD cells significantly reversed the increased activation of NLRP3 and caspase-8 (Fig. S3F). Furthermore, the results demonstrated that the expression of cleaved GSDME decreased after the knockdown of NLRP3, caspase-8, or caspase-3 (Fig. S4A–S4C). It was also found that the levels of NLRP3 and cleaved caspase-8, GSDME, and caspase-3 were lower in the NLRP3 inhibitor MCC950 treatment groups than in the control group (Fig. S4D). To investigate whether APE1 inhibition affects chemotherapy drug-induced cell pyroptosis, we treated APE1-KD or WT NSCLC cells with tumor necrosis factor-alpha (TNF-α) and doxycycline (DOX) to assess the extent of pyroptosis in the cells. The results showed that TNF-α or DOX treatment enhanced the level of cell pyroptosis in the APE1-KD cells compared with those in the control cells (Fig. S5A–S5C). We also used the previously reported small-molecule compound CRT to confirm that APE1 inhibition could promote cell pyroptosis (Fig. S5D–S5H).

We further explored whether pyroptosis triggered by APE1 depletion could suppress tumor progression *in vivo*. C57BL/6 mice were reared for subcutaneously implanted with LLC cells for tumorigenesis. There was no significant difference in the body weight of mice (Fig. S6A). Tumors derived from APE1-knockdown LLC cells exhibited reduced growth and final size compared with those from WT cells; however, this inhibitory effect was significantly abrogated upon NLRP3 knockdown (Fig. 1L and M). The results showed that the mice bearing APE1-KD cells had elevated serum cytokine levels compared with the WT group (Fig. 1N). Notably, NLRP3 knockdown in syngeneic mice reversed the increase in serum IFN-γ observed in the APE1-KD LLC-derived model (Fig. 1N). Furthermore, enhanced immune cell infiltration was observed in APE1-KD LLC-derived tumor tissues compared with both the WT and APE1/NLRP3 double knockdown groups (Fig. S6B). Moreover, knockdown of APE1 in the syngeneic model resulted in higher chemokines and GSDME levels in the tumor tissues compared with the other groups in our experiments (Fig. S6C and S6D). Next, to further mimic the immune environment of the human body, we constructed a humanized mouse model using B-NDG mice (Fig. S7A). The mice were divided into four groups: two groups received tail vein injections of human peripheral blood mononuclear cells (PBMC) to establish a humanized immune system, while the other two groups were injected with saline as a control. Subsequently, WT or APE1 stably knockdown NCI-H460 cells were employed to induce subcutaneous tumor formation in these mice. Finally, four groups of experimental animals were constructed, including non-humanized WT, APE1-KD, humanized WT + PBMC, and APE1-KD + PBMC groups. In the subsequent weeks of immune indicator testing, it was found that the ratio of CD3^+^ T cells to peripheral blood mononuclear cells gradually increased, which indicated the successful construction of humanized mice (Fig. S7B). There was no significant difference in body weight changes among the four groups of mice (Fig. S7C). In immune-reconstituted mice, APE1-KD tumors showed significantly reduced volume compared with controls (Fig. S7D). APE1-KD + PBMC mice exhibited smaller tumors, elevated serum cytokines, and increased immune cell infiltration (Fig. S7E–S7H). Additionally, tumor tissues from the APE1-KD + PBMC mice showed up-regulated NLRP3, GSDME, cleaved caspase-3, and cleaved caspase-8, indicating that APE1 inhibition promotes T cell infiltration and suppresses tumor growth *in vivo* (Fig. S7I–S7L). These results demonstrate that APE1 depletion facilitates T cell infiltration and suppresses tumor growth *in vivo*. In our study, we demonstrate that APE1 depletion in tumor cells induces cell pyroptosis by activating the NLRP3–caspase-3–GSDME pathway, thereby suppressing tumor *in vivo* (Fig. 1O).

In summary, our study reveals that inhibiting the DNA damage repair protein APE1 can induce pyroptosis in tumor cells, releasing inflammatory cytokines and chemokines through the activation of the NLRP3–caspase-3–GSDME pathway *in vitro*. Additionally, APE1 inhibition could promote the infiltration of immune cells into the tumor microenvironment to enhance anti-tumor immunotherapy *in vivo*. Thus, our research offers novel insights and a potential immunotherapy strategy for NSCLC treatment.

## CRediT authorship contribution statement

**Fan Yang:** Writing – review & editing, Writing – original draft, Methodology, Investigation, Data curation. **Kaili Long:** Formal analysis, Data curation. **Yannan Qi:** Investigation. **Yuwei Dai:** Investigation. **Feiyan Pan:** Funding acquisition. **Lingfeng He:** Funding acquisition. **Lindong Yang:** Conceptualization. **Zhigang Guo:** Funding acquisition. **Zhigang Hu:** Writing – review & editing, Funding acquisition, Conceptualization.

## Ethics declaration

All animal experiments were conducted in accordance with protocols approved by the Laboratory Animal Care Committee of Nanjing Normal University and in compliance with NIH guidelines.

## Data availability

RNA sequencing data that support the findings of this study have been deposited in the National Center for Biotechnology Information (NCBI)'s Gene Expression Omnibus (GEO) (GSE220807).

## Funding

This study was supported by the 10.13039/501100001809National Natural Science Foundation of China (No. 32171407, 82373183), Jiangsu Provincial Natural Science Foundation and the 10.13039/501100012246Priority Academic Program Development of Jiangsu Higher Education Institutions.

## Conflict of interests

The authors declared no conflict of interests.
