# Supplementary material for: APE1 inhibition-promoted pyroptosis triggers T-cell infiltration and enhances anti-tumor immunity in NSCLC
Source: Genes Dis. 2025 Aug 14;13(4):101813. doi: 10.1016/j.gendis.2025.101813 (PMC13011028; doi:10.1016/j.gendis.2025.101813)
Supplement: Multimedia component 1 [file mmc1.docx]

**Fig.S1**

**Figure.S1 APE1 depletion promotes tumor cell pyroptosis by activating the Caspase-3-GSDME pathway. (A, B)** Western blot (A) and RT-qPCR (B) for detecting the knockdown efficiency of APE1 in A549, NCI-H460 and LLC cells. **(C)** Tumor cell morphology was observed by transplanted with short hairpin RNA-mediated APE1. Black arrows indicate cell pyroptosis. Scale bar: 50 μm. **(D)** RT-qPCR for detecting the cytokines and chemokines in tumor cells. **(E-H)** ELISA for IFN-γ (E), IL-2 (F), CCL5 (G) and CXCL10 (H) release in supernatants of tumor cells. **(I)** Detection of LDH release in supernatants of tumor cells. **(J)** Hoechst33342/PI fluorescent staining assay for detecting LLC cells membrane integrity. Scale bar: 50 μm. **(K)** Western blot for cell pyroptosis proteins levels in tumors cells, including GSDMA, GSDMB, GSDMC, and GSDMD. **(L)** Western blot for cell pyroptosis proteins levels in LLC cells, including GSDME, Caspase-3, and Caspase-1. Data is represented as mean ± SD of at least three independent experiments. In all plots, *, *P*< 0.05; **, *P* < 0.01; ***, *P* < 0.001.

**Fig.S2**

**Figure.S2 Depletion of APE1 leads to cytoplasmic mtDNA accumulation. (A)** Immunofluorescence (IF) detection of intracellular dsDNA expression in tumor cells by transplanted with short hairpin RNA-mediated APE1. Scale bar: 10 μm. **(B)** Detection of mtDNA expression in tumor cells. **(C)** IF detection of intracellular dsDNA co-localization with Tom20 in LLC cells treated with 10 ng/μL EB after 48h. Scale bar: 50 μm. **(D)** Western blot for cell pyroptosis proteins levels in LLC cells by treated with 10 ng/μL EB after 4 days, including Caspase-3 and GSDME. Data is represented as mean ± SD of at least three independent experiments. In all plots, ***, *P* < 0.001.

**Fig.S3**

**Figure.S3 APE1 depletion triggers tumor cell pyroptosis by activating the NLRP3-Caspase-8 pathway. (A)** IF detection of intracellular dsDNA co-localization with NLRP3 in LLC cells. Scale bar: 10 μm. **(B)** Western blot for cell pyroptosis proteins levels in tumor cells, including AIM2, cGAS, and STING. **(C)** Western blot for cell pyroptosis proteins levels in LLC cells, including NLRP3, Caspase-8 Caspase-3 and GSDME. **(D)** Western blot for cell pyroptosis proteins levels in tumor cells, including IL-1α与IL-1β. **(E)** Representative fluorescent microscopy images of tumor cells that were co-stained for 8-OH-dG, TOM20 and DAPI. Scale bar: 10 μm. **(F)** Western blot for cell pyroptosis proteins levels in tumor cells by treated with 10 ng/μL EB after 4 days, including NLRP3 and Caspase-8. Data is represented as mean ± SD of at least three independent experiments.

**Fig.S4** ****

**Figure.S4 Activation of NLRP3-Caspase-8-Caspase-3 pathway is key to trigger tumor cell pyroptosis. (A-C)** Western blot for cell pyroptosis proteins levels in tumor cells. **(D)** Western blot for cell pyroptosis proteins levels in tumor cells by treated with MCC950 for 12h.

**Fig.S5**

**Figure.S5 Inhibition of APE1 in combination with drug treatment enhances tumor cell pyroptosis. (A, B)** Western blot for cell pyroptosis proteins levels in tumor cells in combination with drugs for 12h, including GSDME and Caspase-3 (A), GSDMD and Caspase-1 (B). **(C)** Detection of LDH release in supernatants of tumor cells in combination with 10 μM/mL DOX or 10 ng/μL for 12h. **(D)** Tumor cell morphology was observed when combined with APE1 inhibitor for 12h, 10 μM/mL CRT. Scale bar: 50 μm. **(E)** Detection of LDH release in supernatants of tumor cells treated with 10 μM/mL CRT combining with 10 μM/mL DOX or 10 ng/μL for 12h. **(F)** Hoechst33342/PI fluorescent staining assay for detecting tumor cells membrane integrity treated with 10 μM/mL CRT combining with 10 μM/mL DOX or 10 ng/μL for 12h. Scale bar: 50 μm. **(G, H)** Western blot for cell pyroptosis proteins levels in tumor cells treated with 10 μM CRT combining with 10 μM/mL DOX or 10 ng/μL for 12h, GSDME and Caspase-3 (G), GSDMD and Caspase-1 (H).

**Fig.S6**

**Figure.S6 APE1 depletion suppresses tumor growth in syngeneic mouse models. (A)** Mice body weights monitored during treatment. **(B)** HE staining for the detection of infiltrating lymphocytes within tumor tissues. Scale bar: 50 μm. **(C)** Tumors were harvested three days post-treatment, subjected to IF analysis for CCL5 and CXCL10. Scale bar: 50 μm. **(D)** Tumors were harvested three days post-treatment, subjected to IF analysis for GSDME. Scale bar: 50 μm. Data is represented as mean ± SD of at least five independent experiments. In all plots, ns, no significance; **, *P* < 0.01.

**Fig.S7**

**Figure.S7 Targeting APE1 promotes tumor regression in humanized mouse models. ((A)** Treatment scheme used in the NCI-H460 APE1-KD xenograft model injected with human PBMC. B-NDG mice were injected with human PBMC in Day0. Then 5×10^6^ NCI-H460 APE1-KD cells injected subcutaneously Day7. **(B)** Detection of human CD3^+^ T-cell expression in peripheral blood of mice with blood collected from tail vein every week in a row mouse models by flow cytometry. **(C)** Mice body weights monitored during treatment. **(D)** Data showing the tumor volume (mm^3^) change trend of mice in 4 different treat groups. **(E)** ELISA results showed the IFN-γ secretion levels in mice blood. **(F)** Detection of the mouse tumor microenvironment for immune infiltration by flow cytometry, including CD45, CD4 and CD8. **(G)** HE staining for the detection of infiltrating lymphocytes within tumor tissues. Scale bar: 50 μm. **(H)** IF showing the expression of CCL5 and CXCL10 in tumor tissue. Scale bar: 50 μm. **(I-L)** IF showing NLRP3 (I), Caspase-8 (J), Caspase-3 (K), GSDME (L) expression in tumor tissue. Scale bar: 50 μm. Data is represented as mean ± SD of at least five independent experiments. In all plots, ***, *P* < 0.001.
