# Supplementary material for: APE1 inhibition-promoted pyroptosis triggers T-cell infiltration and enhances anti-tumor immunity in NSCLC
Source: Genes Dis. 2025 Aug 14;13(4):101813. doi: 10.1016/j.gendis.2025.101813 (PMC13011028; doi:10.1016/j.gendis.2025.101813)
Supplement: Multimedia component 2 [file mmc2.docx]

**Materials and methods**

**Cell culture**

A549 (CCL-185™,) NCI-H460 (HTB-177), and Lewis lung carcinoma (LLC) were obtained from the ATCC. A549^shAPE1^, NCI-H460 ^shAPE1^, and LLC ^shAPE1^ were generated by transfecting the respective cell lines with short hairpin RNA (shRNA) targeting APE1. A549 and NCI-H460 cell lines were cultured in RPMI 1640 medium (Kaiji, Nanjing, China), while LLC cells were maintained in high-glucose DMEM medium (Kaiji, Nanjing, China) containing 10% FBS. All cultures were incubated in a 5% CO2 environment.

**Hoechst 33342/PI double staining**

Tumor cells (5×10⁴) were seeded into a 12-well plate and cultured for 24 hours under standard conditions. After incubation, cells were treated according to the experimental conditions. Subsequently, Hoechst 33342 (1 μg/μL) and PI (1 μg/μL) were added to the cells and incubated at 4°C for 10 minutes for staining. The excess liquid was carefully absorbed with absorbent paper. The slides were then sealed using a mounting medium containing an anti-fade reagent. Finally, cell images were captured using a Laser Scanning Confocal Microscope.

**Western blotting**

The cells were disrupted using SDS lysis buffer supplemented with a protease inhibitor. Each sample underwent sonication three times for 3-second bursts. Following sonication, the proteins were heated at 95°C for 10 minutes to induce denaturation. The cell lysates were subjected to 8 %~12 % SDS-PAGE, followed by transfer onto NC membranes under optimal conditions, then incubated with specific antibodies targeting the following antigens: anti-GSDMA(A10602), GSDMC (A21213), GSDMD (A18281), GSDME(A7432), NLRP3 (A5652), Caspase-8 (A2156), AIM2 (A22874), β-tubulin (AC008), HRP-conjugated mouse anti-Rabbit IgG (AS061), and HRP-conjugated rabbit anti-Goat IgG (AS029) antibodies were from ABclonal Technology; anti-APE1 (10203-1-AP), Caspase-3 (17563-1-AP), cGAS (26416-1-AP), STING (19851-1-AP), and p-STING (#66680-1-Ig) antibodies were from Proteintech; anti-GSDMB (#12885-1-AP) and Caspase-1(#M025280) antibodies were from ABmart Technology.

**Lactate dehydrogenase (LDH) determination**

The release of LDH into the free supernatants, serving as a marker for cell death, was measured using commercial kits ([A020-2-2](http://www.njjcbio.com/products.asp?id=324), Jianchen Bio).

**Enzyme-Linked Immunosorbent Assay (ELISA)**

The tumor cells supernatant was collected to measure the levels of cytokines (IFN-γ, IL-18, and IL-2) by commercial kits (absin, shanghai, China). Additionally, 1 mL of peripheral blood was collected from treated mice and incubated overnight at 4°C. The samples were then centrifuged for 10 minutes at 3,000 rpm for 15 minutes to isolate the serum. Cytokine levels in the serum were measured by commercial kits (absin, shanghai, China).

**RNA isolation and RT-PCR**

Tumor cells were seeded in 35‐mm dishes and cultured for 2 days, or alternatively treated with 10 ng/mL EtBr for 4 days before collection. Total RNA was extracted from cells using the RNA extraction kit (

RM201-02, Vazyme) following the manufacturer’s protocol. Briefly, cells were harvested and lysed using a lysis buffer containing guanidine isothiocyanate, which facilitates the disruption of cell membranes and the release of RNA. After lysis, chloroform was added to separate the aqueous phase containing RNA from the organic phase. RNA quantification and gene expression analysis were carried out by RT-PCR. The relative expression levels of target genes were normalized to the expression of β-actin, which served as an internal control. This normalization ensures the accuracy of gene expression measurements, accounting for variations in RNA quantity and quality across different samples. Primers used for qPCR: human-β-actin-F-5’ ACATCCGCAAAGACCTGTAC3’, human-β-actin-R-5’TGATCTTCATTGTGCTGGGTG3’, mouse-β-actin-F-5’GAAATCGTGCGTGACATCAAAGAG3, mouse-β-actin-R-5’CAATAGTGATGACCTGGCCGTC3’, human-APE1-F-5’ ACATCCGCAAAGACCTGTAC3’, human-APE1-R-5’TGATCTTCATTGTGCTGGGTG3’, mouse-APE1-F-5’ AAACCTCACCCAGTGGCAAA3’, mouse-APE1-R-5’CAATAGTGATGACCTGGCCGTC3’. All samples were tested at least 3 biological replicates.

**qPCR analysis of Cytosolic DNA**

Tumor cells were harvested and suspended in 250 μL of NaCl-HEPES buffer (150 mM NaCl, 20 mM pH 7.4 HEPES, 25 mg/mL digitonin). Following a 5-min incubation on ice, the homogenates underwent an additional 23-min end-over-end incubation for to selectively permeabilize the plasma membrane.. Subsequently, centrifugation at 1,000 g for 5 min at 4 ℃ was carried out. The obtained pellets were reconstituted in 500 μL of 50 mM NaOH and heated at 98°C for 30 minutes to dissolve the DNA. Then 100 μL of 1 M Tris-HCl (pH 8.0) was added to neutralize alkaline conditions and ensure DNA stability. The precipitated total DNA was used as a normalization control for nuclear DNA (nDNA). Carefully transfer the supernatant to a new tube and centrifuge at a higher speed (17,000 × g) for 20 minutes at 4°C to pellet the mitochondria. Resuspend the mitochondrial pellet in a buffer suitable for DNA extraction and proceed with the extraction protocol, typically involving enzymatic digestion and phenol-chloroform extraction or column-based purificationTo measure the level of mitochondrial DNA (mtDNA) in tumor cells by RT-PCR and assess the relative amounts of nuclear DNA (nDNA) and mtDNA. The levels of mtDNA are normalized to nDNA to account for variations in DNA input. The oligonucleotides for qPCR analysis: human-Tert-F-5’ GCATCAGGGGCAAGTCCTAC3’, human-Tert-R-5’ GGGCATAGCTGAGGAAGGTTT 3’, mouse-Tert-F-5’CTAGCTCATGTGTCAAGACCCTCTT3’, mouse-Tert-R-5’ GCCAGCACGTTTCTCTCGTT3’, human-D-Loop-F-5’ATCCCGCACAAGAGTGCTAC3’, human-D-Loop-R-5’AGAGCTCCCGTGAGTGGTTA3’, mouse-D-Loop-F-5’ TCCTCCGTGAAACCAACAA3’, mouse-D-Loop-R-5’ AGCGAGAAGAGGGGCATT3’. All samples were tested at least 3 biological replicates.

**Immunofluorescence**

Seed cells onto glass coverslips in 6-well or 12-well plates and allow them to adhere overnight. Immunofluorescence was employed to analyze the expression of dsDNA, NLRP3, and Tom20 in tumor cells (A549, NCI-H460, and LLC). Fix cells with 4% paraformaldehyde (PFA) in PBS for 15-20 minutes at room temperature. After fixation, wash the cells three times with PBS. Incubate cells with 0.5% Triton X-100 in PBS for 10 minutes at room temperature to permeabilize the cell membranes, allowing the antibodies to enter. After permeabilization, wash cells again with PBS. To 3 % BSA was added to prevent nonspecific binding at room temperature for 1 hour. Each slide was then incubated with appropriate amounts of anti-dsDNA (sc-58749, Santa cuz), anti-NLRP3 (A5652, Abclonal), and anti-Tom20 (A199403, Abclonal) antibodies and placed in a humidified chamber for overnight incubation at 4°C. After incubation, the samples were washed and subsequently incubated with anti-Mouse Alexa Fluor 488 nm and anti-RabbitAlexa Fluor 594 nm for 2 hours at room temperature. The samples were then incubated with DAPI for 15 minutes. Afterward, the slides were washed three times with PBST for 10 minutes each to remove excess DAPI. Mount the coverslips onto glass slides using a mounting medium that contains an anti-fade agent. Visualize the stained cells by Fluorescence microscopy (Nikon, 80I 10-1500X).

**In *vivo* tumor model**

All animal experiments were carried out according to protocols authorized by the Laboratory Animal Care Committee at Nanjing Normal University, in compliance with the NIH guidelines. Four-week-old female B-NDG mice were obtained from GemPharmatech. Human PBMCs were suspended in cold PBS and injected aseptically via the tail vein (100 μL per mouse) for three days. After seven days, tumor cells (5×10⁶ per mouse) were implanted subcutaneously into the right lower leg. The mice were randomly divided into four groups: NCI-H460-WT, NCI-H460-APE1-KD, NCI-H460-WT+PBMC, and NCI-H460-APE1-KD+PBMC.

Similarly, four-week-old female C57BL/6 mice were purchased from the same supplier and acclimated under specific pathogen-free conditions for three days before receiving subcutaneous injections of tumor cells (5×10⁶ per mouse) in the right flank. The mice were randomly assigned to four groups: LLC-WT, LLC-NLRP3-KD, LLC-APE1-KD, and LLC-NLRP3-KD + APE1-KD.

Tumor growth was monitored every three days by measuring tumor length (L) and width (W) with a digital vernier caliper, and tumor volume (V) was determined using the formula V = (L × W²) / 2. Mouse body weight was recorded at the same time points. At the study endpoint, all mice were euthanized via cervical dislocation. Tumors were excised, photographed, weighed, and prepared for paraffin embedding. Blood samples were collected before euthanasia to evaluate cytokine release levels in *vivo*.

**Immunohistochemistry (IHC)**

Tissues were fixed in 4 % paraformaldehyde, embedded in paraffin, and cut into 3 μm sections. After deparaffinization and rehydration, antigen retrieval was performed by incubating the slides in a heat-induced epitope retrieval solution at 95°C for 30 minutes. To minimize non-specific binding, a blocking buffer was applied, followed by incubation with primary antibodies CD45, CD4, and CD8 (Abcam, Grand Island, NY) at 4°C. After washing, the slides were treated with a secondary antibody conjugated to a reporter enzyme, and the signal was visualized using a chromogenic substrate. Finally, the slides were counterstained with hematoxylin, dehydrated, and mounted for observation under a light microscope.

**Statistical analysis**

Statistical analyses were performed using GraphPad Prism 9.0, while flow cytometry data processing and visualization were conducted with cytoFLEX software. A one-sample t-test was used for fold-change comparisons between two groups. One-way ANOVA was applied to compare three or more independent group. Sidak’s post-hoc test for selected pairwise comparisons. Kaplan-Meier survival analysis was assessed using the Log-Rank test to show statistical significance. Data are presented as mean ± SD from a minimum of three independent experiments. In all plots, *, *P*< 0.05; **, *P* < 0.01; ***, *P* < 0.001.
